# Di-μ-chlorido-bis­({9-[(2,6-diisopropyl­phen­yl)imino­meth­yl]anthracen-1-yl}palladium(II))

**DOI:** 10.1107/S1600536808010301

**Published:** 2008-04-23

**Authors:** Jin Zhou, Qibao Wang, Hongjian Sun

**Affiliations:** a School of Chemistry and Chemical Engineering, Shandong University, Shanda Nanlu 27, Jinan 250100, People’s Republic of China

## Abstract

The centrosymmetric title compound, [Pd_2_Cl_2_(C_27_H_26_N)_2_], was obtained by a C—H bond-activation reaction of a Schiff base ligand with Li_2_PdCl_4_ in methanol, and was crystallized from dichloro­methane as orange crystals. The Pd atom displays a slightly distorted square-planar geometry, with the N- and C-atom donors in a *cis* arrangement.

## Related literature

An imine palladacycle crystal structure with a six-membered ring has been determined (Munno *et al.* 1995 ▶). For related literature, see: Dupont *et al.* (2005 ▶).
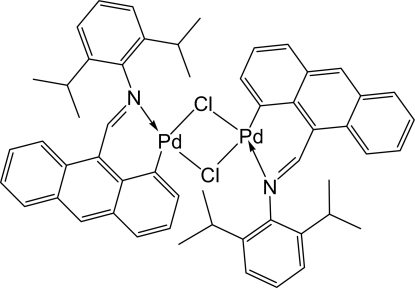


## Experimental

### 

#### Crystal data

[Pd_2_Cl_2_(C_27_H_26_N)_2_]
*M* *_r_* = 1012.68
Monoclinic, 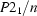

*a* = 12.3002 (4) Å
*b* = 12.9836 (4) Å
*c* = 15.4558 (5) Å
β = 110.364 (2)°
*V* = 2314.04 (13) Å^3^
*Z* = 2
Mo *K*α radiation
μ = 0.93 mm^−1^
*T* = 298 (2) K
0.20 × 0.18 × 0.15 mm

#### Data collection

Bruker SMART APEXII diffractometer
Absorption correction: multi-scan (*SADABS*; Sheldrick, 1996 ▶) *T*_min_ = 0.836, *T*_max_ = 0.873
27723 measured reflections
5320 independent reflections
2428 reflections with *I* > 2σ(*I*)
*R*_int_ = 0.101

#### Refinement

*R*[*F* ^2^ > 2σ(*F* ^2^)] = 0.050
*wR*(*F* ^2^) = 0.131
*S* = 0.94
5320 reflections
271 parameters
H-atom parameters constrained
Δρ_max_ = 0.58 e Å^−3^
Δρ_min_ = −0.54 e Å^−3^

### 

Data collection: *APEX2* (Bruker, 2005 ▶); cell refinement: *APEX2*; data reduction: *SAINT* (Bruker, 1997 ▶); program(s) used to solve structure: *SHELXS97* (Sheldrick, 2008 ▶); program(s) used to refine structure: *SHELXL97* (Sheldrick, 2008 ▶); molecular graphics: *SHELXTL* (Sheldrick, 2008 ▶); software used to prepare material for publication: *SHELXTL*.

## Supplementary Material

Crystal structure: contains datablocks I, global. DOI: 10.1107/S1600536808010301/cs2073sup1.cif

Structure factors: contains datablocks I. DOI: 10.1107/S1600536808010301/cs2073Isup2.hkl

Additional supplementary materials:  crystallographic information; 3D view; checkCIF report

## Figures and Tables

**Fig. 1.**
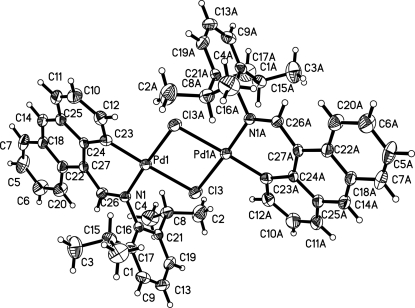
Molecular structure of the title compound. Displacement ellipsoids are drawn at 30% probability level, symmetry code A: 0.5-X, 0.5+Y, 0.5-Z

## supplementary crystallographic information

### Comment

Recently, much attention is being paid to the use of palladacycles as catalyst precursors (Dupont *et al.* 2005). Several imine-palladacycles with six-membered rings were prepared as catalyst precursors (Munno *et al.* 1995). We prepared a new palladacycle with six-membered ring by a C—H bond activation reaction between a Schiff base ligand and Li_2_PdCl_4_ in methanol. In its orange crystals palladium displays a slightly distorted square-planar geometry with the nitrogen and the carbon donors in *cis*-fashion. The length of C26—N1 (1.292 (6) Å) is characteristic for a C=N double bond. The distances of Pd—Cl bond *trans* to the carbon donor are slightly longer (*ca* 0.12 Å) than those *trans* to the imine donor, as expected, because of the *trans* influence. The chelate ring of Pd1—N1—C26—C27—C24—C23 adopts a twisted half-chair conformation, which is due to the presence of three double bonds of N1—C26, C23—C24 and C24—C27 in the ring. The isopropyl groups on the aromatic ring may readily force the *N*-phenyl ring perpendicular to the coordination plane.

### Experimental

Anthracen-9-ylmethylene-(2,6-di-isopropylphenyl)-amine (383 mg, 1.05 mmol) was mixed with Li_2_PdCl_4_ (262 mg, 1 mmol) and NaOAc (86 mg, 1.05 mmol) in 5 ml of methanol. The reaction mixture was stirred for 3 days. The title compound was filtrated and dried under vacuo in the yield of 65% (329 mg). The crystals suitable for X-ray analysis were obtained from a dichloromethane solution by slow evaporation at room temperatures.

### Refinement

All H atoms were fixed geometrically and treated as riding on their parent atoms with C—H = 0.93 Å (aromatic), 0.96 (methyl), 0.98 Å (methine) with *U*_iso_(H) = 1.2 (1.5 for methyl groups) times *U*_eq_(C).

### Crystal data

**Table:**

[Pd_2_Cl_2_(C_27_H_26_N)_2_]	*F*_000_ = 1032
*M**_r_* = 1012.68	*D*_x_ = 1.453 Mg m^−^^3^
Monoclinic, *P*2_1_/*n*	Mo *K*α radiation λ = 0.71073 Å
*a* = 12.3002 (4) Å	Cell parameters from 2187 reflections
*b* = 12.9836 (4) Å	θ = 2.4–18.8º
*c* = 15.4558 (5) Å	µ = 0.93 mm^−^^1^
β = 110.364 (2)º	*T* = 298 (2) K
*V* = 2314.04 (13) Å^3^	Block, orange
*Z* = 2	0.20 × 0.18 × 0.15 mm

### Data collection

**Table:**

Bruker SMART APEXII diffractometer	5320 independent reflections
Radiation source: fine-focus sealed tube	2428 reflections with *I* > 2σ(*I*)
Monochromator: graphite	*R*_int_ = 0.101
*T* = 298(2) K	θ_max_ = 27.5º
φ and ω scans	θ_min_ = 1.8º
Absorption correction: multi-scan(SADABS; Sheldrick, 1996)	*h* = −16→14
*T*_min_ = 0.836, *T*_max_ = 0.873	*k* = −16→16
27723 measured reflections	*l* = −19→20

### Refinement

**Table:**

Refinement on *F*^2^	Secondary atom site location: difference Fourier map
Least-squares matrix: full	Hydrogen site location: inferred from neighbouring sites
*R*[*F*^2^ > 2σ(*F*^2^)] = 0.050	H-atom parameters constrained
*wR*(*F*^2^) = 0.131	*w* = 1/[σ^2^(*F*_o_^2^) + (0.0537*P*)^2^] where *P* = (*F*_o_^2^ + 2*F*_c_^2^)/3
*S* = 0.94	(Δ/σ)_max_ < 0.001
5320 reflections	Δρ_max_ = 0.58 e Å^−^^3^
271 parameters	Δρ_min_ = −0.54 e Å^−^^3^
Primary atom site location: structure-invariant direct methods	Extinction correction: none

### Special details

**Table:**

Geometry. All e.s.d.'s (except the e.s.d. in the dihedral angle between two l.s. planes) are estimated using the full covariance matrix. The cell e.s.d.'s are taken into account individually in the estimation of e.s.d.'s in distances, angles and torsion angles; correlations between e.s.d.'s in cell parameters are only used when they are defined by crystal symmetry. An approximate (isotropic) treatment of cell e.s.d.'s is used for estimating e.s.d.'s involving l.s. planes.

### Fractional atomic coordinates and isotropic or equivalent isotropic displacement parameters (Å^2^)

**Table:**

	*x*	*y*	*z*	*U*_iso_*/*U*_eq_	
Pd1	0.40636 (3)	0.49507 (3)	0.06810 (3)	0.04914 (17)	
C27	0.1926 (4)	0.4388 (4)	0.1521 (3)	0.0411 (13)	
Cl3	0.57537 (14)	0.40623 (14)	0.05309 (12)	0.0827 (6)	
N1	0.3915 (3)	0.3993 (3)	0.1645 (3)	0.0395 (10)	
C26	0.3002 (4)	0.3840 (4)	0.1860 (3)	0.0426 (13)	
H26A	0.3049	0.3316	0.2282	0.051*	
C25	0.0810 (4)	0.5956 (4)	0.0888 (4)	0.0485 (14)	
C24	0.1851 (4)	0.5348 (4)	0.1064 (3)	0.0400 (13)	
C23	0.2731 (4)	0.5759 (4)	0.0773 (3)	0.0466 (14)	
C22	0.0973 (4)	0.4010 (4)	0.1764 (4)	0.0496 (14)	
C21	0.5028 (4)	0.2374 (4)	0.1872 (3)	0.0429 (13)	
C20	0.1011 (5)	0.3082 (5)	0.2267 (4)	0.0666 (18)	
H20A	0.1682	0.2684	0.2455	0.080*	
C19	0.5995 (5)	0.1817 (4)	0.2388 (4)	0.0545 (16)	
H19A	0.6084	0.1145	0.2217	0.065*	
C18	−0.0089 (5)	0.4584 (5)	0.1491 (4)	0.0492 (14)	
C17	0.5775 (4)	0.3840 (4)	0.2893 (4)	0.0447 (13)	
C16	0.4925 (4)	0.3376 (4)	0.2149 (3)	0.0414 (13)	
C15	0.5675 (5)	0.4939 (4)	0.3150 (4)	0.0560 (15)	
H15A	0.5160	0.5288	0.2597	0.067*	
C14	−0.0114 (5)	0.5520 (5)	0.1076 (4)	0.0573 (16)	
H14A	−0.0803	0.5892	0.0907	0.069*	
C13	0.6832 (5)	0.2241 (5)	0.3152 (4)	0.0572 (16)	
H13A	0.7471	0.1854	0.3500	0.069*	
C12	0.2641 (5)	0.6770 (5)	0.0496 (4)	0.0666 (18)	
H12A	0.3250	0.7058	0.0353	0.080*	
C11	0.0777 (6)	0.6980 (5)	0.0583 (4)	0.0668 (18)	
H11A	0.0115	0.7372	0.0498	0.080*	
C10	0.1665 (6)	0.7400 (5)	0.0415 (5)	0.077 (2)	
H10A	0.1651	0.8090	0.0249	0.092*	
C9	0.6706 (5)	0.3239 (5)	0.3391 (4)	0.0572 (16)	
H9A	0.7269	0.3521	0.3907	0.069*	
C8	0.4119 (5)	0.1866 (4)	0.1027 (4)	0.0578 (16)	
H8A	0.3638	0.2416	0.0650	0.069*	
C7	−0.1044 (5)	0.4211 (5)	0.1699 (5)	0.0684 (19)	
H7A	−0.1740	0.4571	0.1494	0.082*	
C6	0.0057 (5)	0.2770 (5)	0.2479 (5)	0.081 (2)	
H6A	0.0096	0.2172	0.2819	0.097*	
C5	−0.0964 (6)	0.3348 (6)	0.2185 (6)	0.087 (2)	
H5A	−0.1599	0.3128	0.2331	0.105*	
C4	0.3321 (5)	0.1145 (5)	0.1306 (5)	0.095 (2)	
H4A	0.2759	0.0854	0.0763	0.142*	
H4B	0.2931	0.1525	0.1645	0.142*	
H4C	0.3771	0.0603	0.1687	0.142*	
C3	0.5130 (7)	0.5030 (5)	0.3888 (5)	0.101 (2)	
H3A	0.4396	0.4681	0.3688	0.152*	
H3B	0.5015	0.5744	0.3994	0.152*	
H3C	0.5633	0.4723	0.4450	0.152*	
C2	0.4703 (6)	0.1313 (6)	0.0437 (5)	0.103 (3)	
H2A	0.5176	0.1792	0.0252	0.154*	
H2B	0.4122	0.1033	−0.0102	0.154*	
H2C	0.5180	0.0766	0.0787	0.154*	
C1	0.6817 (6)	0.5514 (5)	0.3466 (5)	0.095 (2)	
H1A	0.7167	0.5465	0.3001	0.143*	
H1B	0.7326	0.5217	0.4030	0.143*	
H1C	0.6682	0.6225	0.3567	0.143*	

### Atomic displacement parameters (Å^2^)

**Table:**

	*U*^11^	*U*^22^	*U*^33^	*U*^12^	*U*^13^	*U*^23^
Pd1	0.0378 (2)	0.0662 (3)	0.0482 (3)	0.0092 (2)	0.02099 (18)	0.0192 (2)
C27	0.040 (3)	0.051 (4)	0.037 (3)	0.002 (3)	0.018 (3)	−0.004 (3)
Cl3	0.0636 (10)	0.1143 (14)	0.0903 (13)	0.0401 (10)	0.0520 (10)	0.0621 (11)
N1	0.034 (2)	0.043 (3)	0.042 (3)	0.0013 (19)	0.014 (2)	0.007 (2)
C26	0.038 (3)	0.043 (3)	0.049 (3)	0.003 (2)	0.017 (3)	0.010 (3)
C25	0.043 (3)	0.060 (4)	0.044 (3)	0.004 (3)	0.018 (3)	−0.004 (3)
C24	0.037 (3)	0.053 (4)	0.033 (3)	0.003 (2)	0.016 (2)	−0.003 (2)
C23	0.051 (3)	0.045 (4)	0.044 (3)	0.006 (3)	0.016 (3)	0.007 (3)
C22	0.045 (3)	0.056 (4)	0.053 (4)	0.001 (3)	0.022 (3)	−0.002 (3)
C21	0.039 (3)	0.059 (4)	0.036 (3)	0.005 (3)	0.020 (3)	0.010 (3)
C20	0.045 (4)	0.068 (4)	0.088 (5)	−0.004 (3)	0.026 (3)	0.009 (4)
C19	0.048 (4)	0.049 (4)	0.074 (5)	0.010 (3)	0.031 (3)	0.012 (3)
C18	0.043 (3)	0.059 (4)	0.050 (4)	0.006 (3)	0.021 (3)	−0.005 (3)
C17	0.038 (3)	0.060 (4)	0.038 (3)	−0.005 (3)	0.015 (3)	0.010 (3)
C16	0.035 (3)	0.058 (4)	0.038 (3)	0.007 (3)	0.022 (3)	0.016 (3)
C15	0.056 (3)	0.057 (4)	0.050 (3)	−0.008 (3)	0.012 (3)	−0.002 (3)
C14	0.052 (4)	0.070 (4)	0.051 (4)	0.015 (3)	0.020 (3)	−0.008 (3)
C13	0.043 (3)	0.067 (4)	0.059 (4)	0.004 (3)	0.014 (3)	0.022 (3)
C12	0.071 (4)	0.074 (5)	0.064 (4)	0.009 (4)	0.036 (4)	0.013 (4)
C11	0.081 (5)	0.062 (4)	0.070 (5)	0.024 (4)	0.042 (4)	0.007 (4)
C10	0.102 (6)	0.052 (4)	0.090 (5)	0.021 (4)	0.051 (5)	0.023 (4)
C9	0.049 (4)	0.071 (5)	0.045 (4)	−0.010 (3)	0.009 (3)	0.010 (3)
C8	0.055 (4)	0.055 (4)	0.057 (4)	0.004 (3)	0.012 (3)	−0.003 (3)
C7	0.044 (4)	0.079 (5)	0.089 (5)	−0.001 (3)	0.033 (4)	−0.024 (4)
C6	0.065 (4)	0.077 (5)	0.109 (6)	−0.013 (4)	0.041 (4)	0.014 (4)
C5	0.052 (4)	0.091 (6)	0.139 (7)	−0.011 (4)	0.058 (5)	−0.012 (5)
C4	0.068 (5)	0.108 (6)	0.096 (6)	−0.031 (4)	0.013 (4)	−0.006 (5)
C3	0.130 (7)	0.079 (5)	0.116 (6)	−0.017 (5)	0.070 (6)	−0.033 (5)
C2	0.079 (5)	0.122 (7)	0.097 (6)	0.011 (4)	0.018 (5)	−0.048 (5)
C1	0.085 (6)	0.072 (5)	0.124 (7)	−0.022 (4)	0.030 (5)	−0.003 (5)

### Geometric parameters (Å, °)

**Table:**

Pd1—C23	1.992 (5)	C15—C3	1.516 (8)
Pd1—N1	1.997 (4)	C15—H15A	0.9800
Pd1—Cl3^i^	2.3440 (15)	C14—H14A	0.9300
Pd1—Cl3	2.4580 (15)	C13—C9	1.370 (7)
C27—C24	1.420 (7)	C13—H13A	0.9300
C27—C22	1.436 (7)	C12—C10	1.421 (8)
C27—C26	1.431 (6)	C12—H12A	0.9300
Cl3—Pd1^i^	2.3440 (15)	C11—C10	1.326 (8)
N1—C26	1.292 (6)	C11—H11A	0.9300
N1—C16	1.456 (6)	C10—H10A	0.9300
C26—H26A	0.9300	C9—H9A	0.9300
C25—C11	1.405 (7)	C8—C2	1.523 (8)
C25—C14	1.388 (7)	C8—C4	1.522 (8)
C25—C24	1.448 (7)	C8—H8A	0.9800
C24—C23	1.413 (7)	C7—C5	1.333 (8)
C23—C12	1.374 (7)	C7—H7A	0.9300
C22—C20	1.426 (7)	C6—C5	1.396 (8)
C22—C18	1.434 (7)	C6—H6A	0.9300
C21—C16	1.388 (7)	C5—H5A	0.9300
C21—C19	1.383 (7)	C4—H4A	0.9600
C21—C8	1.542 (7)	C4—H4B	0.9600
C20—C6	1.384 (7)	C4—H4C	0.9600
C20—H20A	0.9300	C3—H3A	0.9600
C19—C13	1.383 (7)	C3—H3B	0.9600
C19—H19A	0.9300	C3—H3C	0.9600
C18—C14	1.370 (7)	C2—H2A	0.9600
C18—C7	1.407 (8)	C2—H2B	0.9600
C17—C9	1.378 (7)	C2—H2C	0.9600
C17—C16	1.394 (7)	C1—H1A	0.9600
C17—C15	1.498 (7)	C1—H1B	0.9600
C15—C1	1.514 (7)	C1—H1C	0.9600
			
C23—Pd1—N1	89.05 (19)	C9—C13—C19	119.1 (5)
C23—Pd1—Cl3^i^	94.53 (16)	C9—C13—H13A	120.5
N1—Pd1—Cl3^i^	174.64 (12)	C19—C13—H13A	120.5
C23—Pd1—Cl3	176.05 (17)	C23—C12—C10	123.5 (6)
N1—Pd1—Cl3	94.50 (12)	C23—C12—H12A	118.3
Cl3^i^—Pd1—Cl3	82.05 (5)	C10—C12—H12A	118.3
C24—C27—C22	120.8 (5)	C10—C11—C25	121.7 (6)
C24—C27—C26	120.7 (4)	C10—C11—H11A	119.2
C22—C27—C26	118.0 (5)	C25—C11—H11A	119.2
Pd1^i^—Cl3—Pd1	97.95 (5)	C11—C10—C12	118.3 (6)
C26—N1—C16	115.7 (4)	C11—C10—H10A	120.8
C26—N1—Pd1	127.0 (3)	C12—C10—H10A	120.8
C16—N1—Pd1	117.3 (3)	C17—C9—C13	122.3 (5)
N1—C26—C27	127.2 (5)	C17—C9—H9A	118.8
N1—C26—H26A	116.4	C13—C9—H9A	118.8
C27—C26—H26A	116.4	C2—C8—C4	111.4 (6)
C11—C25—C14	121.7 (6)	C2—C8—C21	110.8 (5)
C11—C25—C24	119.9 (5)	C4—C8—C21	111.8 (5)
C14—C25—C24	118.3 (5)	C2—C8—H8A	107.5
C27—C24—C23	124.4 (5)	C4—C8—H8A	107.5
C27—C24—C25	118.2 (5)	C21—C8—H8A	107.5
C23—C24—C25	117.4 (5)	C5—C7—C18	121.0 (6)
C12—C23—C24	118.2 (5)	C5—C7—H7A	119.5
C12—C23—Pd1	117.4 (4)	C18—C7—H7A	119.5
C24—C23—Pd1	124.4 (4)	C20—C6—C5	120.5 (6)
C27—C22—C20	123.8 (5)	C20—C6—H6A	119.8
C27—C22—C18	119.3 (5)	C5—C6—H6A	119.8
C20—C22—C18	116.9 (5)	C7—C5—C6	121.0 (6)
C16—C21—C19	117.7 (5)	C7—C5—H5A	119.5
C16—C21—C8	123.0 (5)	C6—C5—H5A	119.5
C19—C21—C8	119.2 (5)	C8—C4—H4A	109.5
C6—C20—C22	120.4 (6)	C8—C4—H4B	109.5
C6—C20—H20A	119.8	H4A—C4—H4B	109.5
C22—C20—H20A	119.8	C8—C4—H4C	109.5
C13—C19—C21	121.3 (5)	H4A—C4—H4C	109.5
C13—C19—H19A	119.4	H4B—C4—H4C	109.5
C21—C19—H19A	119.4	C15—C3—H3A	109.5
C14—C18—C7	121.9 (6)	C15—C3—H3B	109.5
C14—C18—C22	117.9 (5)	H3A—C3—H3B	109.5
C7—C18—C22	120.1 (6)	C15—C3—H3C	109.5
C9—C17—C16	117.1 (5)	H3A—C3—H3C	109.5
C9—C17—C15	121.4 (5)	H3B—C3—H3C	109.5
C16—C17—C15	121.5 (5)	C8—C2—H2A	109.5
C21—C16—C17	122.4 (5)	C8—C2—H2B	109.5
C21—C16—N1	120.2 (5)	H2A—C2—H2B	109.5
C17—C16—N1	117.4 (5)	C8—C2—H2C	109.5
C17—C15—C1	113.7 (5)	H2A—C2—H2C	109.5
C17—C15—C3	111.9 (5)	H2B—C2—H2C	109.5
C1—C15—C3	108.8 (5)	C15—C1—H1A	109.5
C17—C15—H15A	107.4	C15—C1—H1B	109.5
C1—C15—H15A	107.4	H1A—C1—H1B	109.5
C3—C15—H15A	107.4	C15—C1—H1C	109.5
C18—C14—C25	125.0 (6)	H1A—C1—H1C	109.5
C18—C14—H14A	117.5	H1B—C1—H1C	109.5
C25—C14—H14A	117.5		

Symmetry codes: (i) −*x*+1, −*y*+1, −*z*.
